# Deciphering B cell Maturation Dynamics in Hyper-IgM Syndromes

**DOI:** 10.1007/s10875-026-02046-6

**Published:** 2026-07-09

**Authors:** Hande Üçler Çınar, Murat Cansever, Şerife Erdem, Abdullah Arık, Ahmet Eken

**Affiliations:** 1 Division of Pediatric Allergy and Immunology, Kayseri City Hospital, Kayseri, Türkiye; 2 Department of Immunology, Faculty of Medicine, Kirsehir Ahi Evran University, Kirsehir, Türkiye; 3 Department of Medical Biology, Faculty of Medicine, Erciyes University, Kayseri, Türkiye; 4 Genome and Stem Cell Center (GENKÖK), Kayseri, Türkiye; 5 Department of Immunology and Microbiology, School of Medicine, University of Colorado Anschutz Medical Campus, Aurora, CO USA

**Keywords:** Hyper-IgM syndrome, B cell maturation, CD40L, AICDA, Class-switch recombination

## Abstract

**Purpose:**

Hyper-IgM syndromes (HIGM) are primary immunodeficiencies characterized by defective class-switch recombination (CSR) and impaired humoral immunity. While genetic causes such as *CD40L* and *AICDA* mutations are well established, a detailed comparison of B cell maturation dynamics across HIGM subtypes remains limited. To comprehensively characterize B cell immunophenotypes and functional responses in patients with HIGM and to delineate mutation-specific differences in B cell maturation and proliferation.

**Method:**

Four patients with genetically confirmed HIGM (one *CD40L* and three homozygous *AICDA* mutations, c.70C>T; p.R24W) and age- and sex-matched healthy controls were studied. Peripheral blood mononuclear cells were analyzed by multiparameter flow cytometry to define B cell subsets based on CD19, CD20, CD24, CD27, CD38, IgD, and IgM expression. B cell proliferation was assessed following CpG stimulation.

**Results:**

All patients exhibited a marked reduction of class-switched memory B cells (CD27⁺IgD⁻) and accumulation of naive B cells (CD27⁻IgD⁺), consistent with defective CSR. The *CD40L*-deficient patient demonstrated profound depletion of plasmablasts and precursor skewing, reflecting failure of germinal center formation. In contrast, *AID* patients showed preserved CD27 expression with variable expansion of transitional and plasmablast populations, suggesting intact T cell–dependent activation but intrinsic failure of CSR. Functional assays revealed heterogeneous proliferative responses in *CD40L* deficiency and *AICDA*-deficient patient, but impaired proliferation in another *AICDA*-deficient individual, highlighting inter-individual variability.

**Conclusion:**

Detailed immunophenotyping reveals distinct B cell maturation arrest points in *CD40L*- versus *AICDA*-associated HIGM. Flow cytometric analysis of B cell subsets provides valuable insights into disease mechanisms, supports differential diagnosis, and may inform clinical monitoring and therapeutic decision-making in HIGM.

## Introduction

Hyper IgM Syndrome (HIGM), first described by Rosen et al. in 1961, is a primary immunodeficiency characterized by normal or elevated serum IgM and markedly low serum IgA, IgG, and IgE levels [1]. This aberrant immunoglobulin profile results from a fundamental defect in isotype switching, a critical process for the development of effective humoral immunity. Defects occur during in the class switch recombination (CSR) and somatic hypermutation (SHM) steps of mature B cell development due to various genetic mutations [2].

Since the initial identification of mutations in the CD40 ligand (CD40L)-encoding gene in 1992, genetic heterogeneity has been increasingly recognized in HIGM cases [3, 4]. The most common form of HIGM is the X-linked type (HIGM type 1), which arises from mutations in the CD40L gene. In contrast, autosomal recessive forms, such as those caused by mutations in the AICDA or CD40 genes [5], are more prevalent in populations with high rates of consanguineous marriages, including Turkiye. In Type 1 HIGM (CD40L or CD154 deficiency), the defect lies in the lack of T-cell-mediated signaling prevents the interaction between CD40L on activated T cells and CD40 on B cells [6]. Conversely, Activation-Induced Cytidine Deaminase (AID) plays a central role in both SHM and CSR. At the molecular level, AID initiates these processes by converting deoxycytidine (dC) into deoxyuridine (dU) within targeted DNA strands [7]. This deamination acts as the primary physiological trigger for genomic remodeling, occurring at a pre-cleavage stage to regulate both point mutations in the V region and recombination in the S region [8].

The development and maturation of B lymphocytes are meticulously orchestrated processes, characterized by the sequential and precise modulation of a plethora of cell surface markers. These markers serve not only as identifiers for distinct B cell subsets but also as pivotal mediators of signal transduction and intercellular communication. In HIGM, disruptions in isotype switching profoundly impact B cell maturation, leading to alterations in the expression patterns of key surface markers.

This study aims to comprehensively characterize the B cell compartment in patients with HIGM, focusing on the expression profiles of CD19, CD20, CD24, CD27, CD38, and the immunoglobulin isotypes IgD and IgM. By employing flow cytometry analysis, we seek to delineate the quantitative and qualitative alterations in B cell subpopulations in HIGM patients, providing a deeper understanding of the immunopathogenesis of this complex disorder. This study will contribute to a more refined characterization of the B cell immunophenotype in HIGM, potentially leading to improved diagnostic and therapeutic strategies.

## Materials and Methods

Patients followed with a diagnosis of HIGM at Department of Kayseri City Hospital Pediatric Allergy and Clinical Immunology were retrospectively screened using their ICD codes. The study cohort consisted of four patients diagnosed with Hyper-IgM (HIGM) syndrome who presented to our center for scheduled routine follow-up and clinical management. To establish a comparative baseline, four healthy controls were included (age range: 4–17 years; 2 males, 2 females), each individually matched for age and sex to a corresponding patient. These control individuals had no history of recurrent infections or chronic diseases, and neither the individuals nor their families had a history of primary or secondary immunodeficiency. Comprehensive information regarding the study objectives was provided to all participants and their legal guardians; subsequently, written informed consent was obtained from the parents of the patients and from the control subjects prior to their enrollment in the study. The study was conducted in accordance with the 2013 revision of the Declaration of Helsinki and was implemented by the Kayseri City Education and Research Hospital Local Ethics Committee with the approval decision numbered 235 dated 05.11.2024.

### PBMC Isolation

Peripheral blood mononuclear cells (PBMCs) were isolated from venous blood samples of patients and age/sex-matched healthy individuals using Ficoll-Paque density gradient centrifugation. Briefly, blood samples were diluted 1:1 with PBS and carefully layered onto Ficoll-Paque (GE Healthcare). Samples were centrifuged at 400 g for 30 min, and the mononuclear cell layer was carefully collected. Cells were washed twice with PBS and suspended in RPMI-1640 culture medium supplemented with 10% FBS. Cell concentration was determined using a hemocytometer and used for phenotyping and proliferation assays.

### B Cell Surface Staining

The gating strategy followed a strict hierarchical approach: lymphocytes were first identified by FSC-A/SSC-A characteristics, debris excluded, followed by singlet selection to exclude doublets, and finally gated on CD19 + to define the total B cell population. For the identification of B cell subpopulations, isolated PBMCs were stained with surface markers after Fc receptor blocking: CD19-FITC, CD20-BV510, CD24-PE, CD27-APC, CD38-PE-Cy7, IgD-PE-Dazzle, CD138-APC-Cy7. After staining, cells were incubated at 4 °C for 30 min, washed with PBS, and prepared for flow cytometry analysis. Analyses were performed using a BD FACSARIA III device, and data were evaluated using FlowJo software.

### B Cell Proliferation Assay

To evaluate B cell proliferation, isolated PBMCs were seeded in 96-well plates at 2 × 10⁵ cells/well and cultured in RPMI-1640 + 10% FBS medium. Cells were stimulated with CpG (20 µg/mL) and incubated for 5 days. Proliferation analysis was performed using: Control group (unstimulated, no CpG), and Experimental group (stimulated with CpG, 20 µg/mL CpG added). At the end of the incubation period, cells were stained with PE-conjugated CD19 antibody and incubated at 4 °C for 30 min. After washing with PBS, cells were analyzed by flow cytometry. Proliferation rates were statistically compared.

### Statistical Analysis Methods

Data analysis was performed by first testing the assumption of normal distribution. The normality of the groups was assessed using the Shapiro-Wilk test. Parametric tests were used for normally distributed data, and non-parametric tests were used for non-normally distributed data. One-way analysis of variance (ANOVA) was used to compare multiple groups. Tukey’s multiple comparison test was performed to determine the source of differences between groups after ANOVA. Statistical Significance was indicated as follows: *p* < 0.05 values were considered statistically significant. p values were expressed with asterisk symbols: *p* < 0.05 → * *p* < 0.01 → ** *p* < 0.001 → *** *p* < 0.0001 → ****All analyses were performed using GraphPad Prism 9 software.

## Result

### Demographic and Clinical Characteristics

The study included 4 patients with a mean age of 8.75 years (range: 4–17), comprising 2 males and 2 females. All patients in the study group shared a common clinical history of recurrent respiratory tract infections (RTIs). Patient 2 (Pt 2), who underwent an adenotonsillectomy at the age of three due to chronic infections, was also followed by the pediatric oncology department for persistent recurrent cervical lymphadenopathy. Following her clinical evaluation, her sister (Pt 3) was screened due to a history of recurrent upper RTIs, leading to her subsequent diagnosis. The family history was notable for third-degree consanguinity between the parents and a maternal relative receiving intravenous immunoglobulin (IVIG) therapy for an unknown immunodeficiency. The cohort included two sisters (Pt 2 and Pt 3) from a consanguineous family, while Pt 1 and Pt 4 were from unrelated, distinct familial backgrounds. All patients exhibited a laboratory profile consistent with Hyper IgM Syndrome (HIGM), characterized by significantly low serum IgG and IgA levels. The Pt1 carried a hemizygous mutation in CD40L (c.346 + 1G > T), a variant functionally characterized by Xue Gong et al. in a patient with defective CD40L expression on activated T cells [9]. The Pt 2, Pt3 and Pt4 presented with a homozygous AICDA mutation (c.70C>T, p.R24W) which was historically documented as a causative HIGM2 mutation in patients of Turkish origin in the seminal study by Revy et al. [10]. Clinical, demographic and laboratory characteristics of patients are shown in Table 1.

**Table 1:** Clinical, demographic and laboratory characteristics of patients

	Pt1	Pt 2	Pt 3	Pt 4
Age/Sex	4, Male	6, Female	8, Female	17, Male
CBC				
Hemoglobin (g/dl)	11.3	11.2	13.8	10.8
WBC (/mm^3^)	8390	7510	6830	8470
ANS (/mm^3^)	2290	3940	3130	3730
Lymphocyte (/mm^3^)	5280	2729	2930	3790
Lymphocytes subsets				
CD3	%70 (56–75)	%72.2 (56–75)	%71 (60–76)	%70 (56–84)
CD4	%51 (28–47)	%39.8 (28–47)	%33 (31–47)	%42 (31–52)
CD8	%14 (16–30)	%24.9 (16–30)	%31 (18–35)	%25 (18–35)
CD16 + 56	%7 (4–17)	%7.2 (4–17)	%11 (4–17)	%8 (3–22)
CD19	%22 (14–33)	%17.8 (14–33)	%16 (12–27)	%20 (6–23)
IgG (mg/dl)	18 (457–1120)	18 (483–1580)	< 0.3 (483–1580)	454 (688–2430)
IgA (mg/dl)	1 (35.7–192)	15 (44.8–276)	1 (44.8–276)	8 (46.3–385)
IgM (mg/dl)	240 (58.7–198)	2453 (50.3–242)	904 (50.3–242)	661 (60.7–323)
IgE (mg/dl)	0.2	0.2		
Gene, zygosity, mutation	*CD40L*, Hemizygous c.346 + 1G > T	Homozygous *AICDA* mutation c.70C>T (p.R24W)		
Complication		Lymphadenopathy, tonsillectomy		

*WBC* White blood count, *ANS* absolute neutrophil count

Serum Ig age-specific reference values are shown in parentheses [11]

### B Cell Subsets Distribution and CSR Defects

**Fig. 1:**
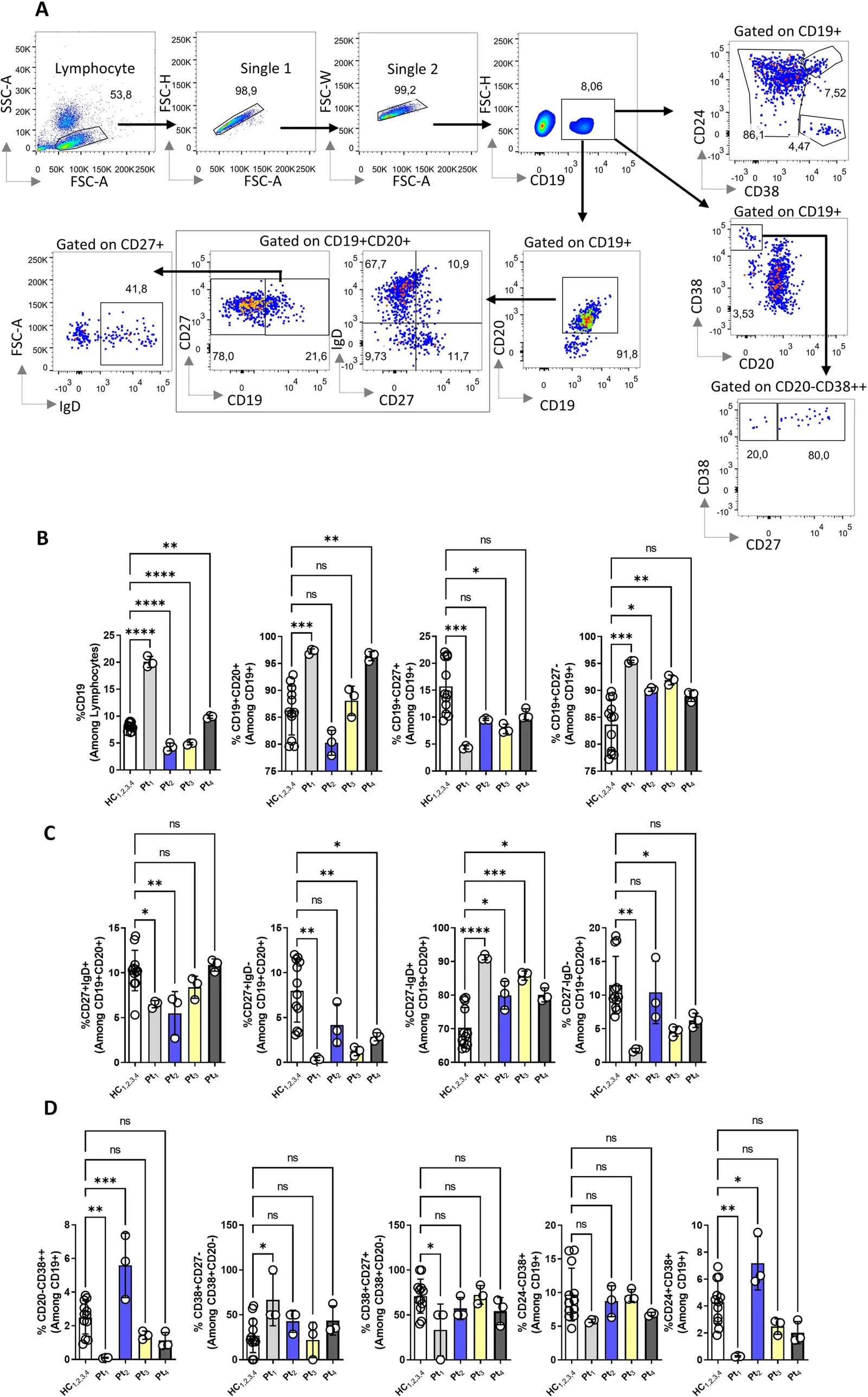
Immunophenotypic analysis of B cell subsets across patients (Pt1–Pt4) and healthy controls (HC). Bars represent mean values, and error bars indicate standard deviation (SD). Each circle represents an individual technical replicate for both patients (Pt 1–4) and their respective age- and sex-matched healthy controls (HC 1–4). Panel (**A**) shows gate strategy for flow cytometry. Panel (**B**) displays total (CD19^+^), CD20^+^, memory (CD27^+^) and naive (CD27^-^) B cell frequencies. Panel (**C**) shows memory and naive B cell distributions based on IgD and CD27 expression. Panel (**D**) illustrates B cell differentiation stages: CD19^+^CD20^-^CD38^+ +^ (Antibody-Secreting Cells (ASCs), include plasmablasts (PBs) and plasma cells (PCs)); CD19^+^CD20^-^CD38^+^CD27^-^ B cells primarily represent transitional cell; CD19 + CD20-CD38^+^CD27^+^ B cells primarily represent Plasma Cells (PCs) or their precursors the Plasmablasts (PBs); CD19^+^CD24^-^CD38^+^) represent plasmablasts, and (CD19^+^CD24^+^CD38^+^) represent immature transitional B cells. Statistical significance was determined using ANOVA and indicated as: *p < 0.05, **p < 0.01, ***p < 0.001, ****p < 0.0001; ns: not significant

The immunophenotypic analysis of patient B cell subsets revealed distinct profiles compared to healthy controls (HC). Figure 1 provides a comprehensive immunophenotypic map comparing the patient group with age- and sex-matched healthy controls (Fig. 1A, gate strategy), detailing B cell development from the total reservoir and naive-to-memory distribution (Fig. 1B), through class-switch recombination (CSR) efficiency at the isotype level (Fig. 1C), to the terminal differentiation of transitional cells into antibody-secreting plasmablasts (Fig. 1D). In terms of total CD19⁺ B cells, Pt1 showed a significant increase, whereas Pt2, Pt3, and Pt4 exhibited a decrease. Specifically, an analysis of the CD19^+^ B cell compartment revealed a significant expansion of the CD19^+^ CD20^+^ population in Patients 1 and 4. Analysis of B cell maturation revealed that while the frequency of CD27^+^ memory B cells was decreased across all patients, this reduction achieved statistical significance in Pt1 and Pt3 (*p* < 0.001 and *p* < 0.05, respectively), although CD27^−^ naive B cells were expanded in the entire cohort, the increase reached statistical significance exclusively in Pt1, Pt2, and Pt3 (*p* < 0.001; *p* < 0.05 and *p* < 0.01, respectively).

Further characterization of the CD19⁺CD20⁺ compartment using CD27 and IgD markers indicated that CD27⁺IgD⁺ cells were significantly reduced in Pt1 and Pt2 (*p* < 0.05 and *p* < 0.01, respectively). Similarly, switched memory cells (CD27⁺IgD⁻) were decreased in Pt1, Pt3, and Pt4, and double-negative cells (CD27⁻IgD⁻) were lower in Pt1 and Pt3. Conversely, CD27⁻IgD⁺ cells were significantly increased across all patients (Pt1–Pt4). Analysis of B cell differentiation stages revealed a significant depletion of CD20⁻CD38⁺⁺ plasmablasts in Pt1 (*p* < 0.01), whereas a marked expansion of this population was observed in Pt2 (*p* < 0.001). Sub-analysis within the CD38⁺CD20⁻ compartment showed a significant increase in the frequency of CD27⁻ precursor B cells in Pt1 (*p* < 0.05), contrasted by a downward trend in CD27⁺ plasma B cells (*p* < 0.05). Furthermore, regarding transitional B cells (CD24⁺CD38⁺), Pt1 exhibited a significant reduction compared to the healthy control group (*p* < 0.01), while Pt2 showed a statistically significant elevation (*p* < 0.05).

### B Cell Functional Analysis: CpG Stimulation and Proliferation

**Fig. 2:**
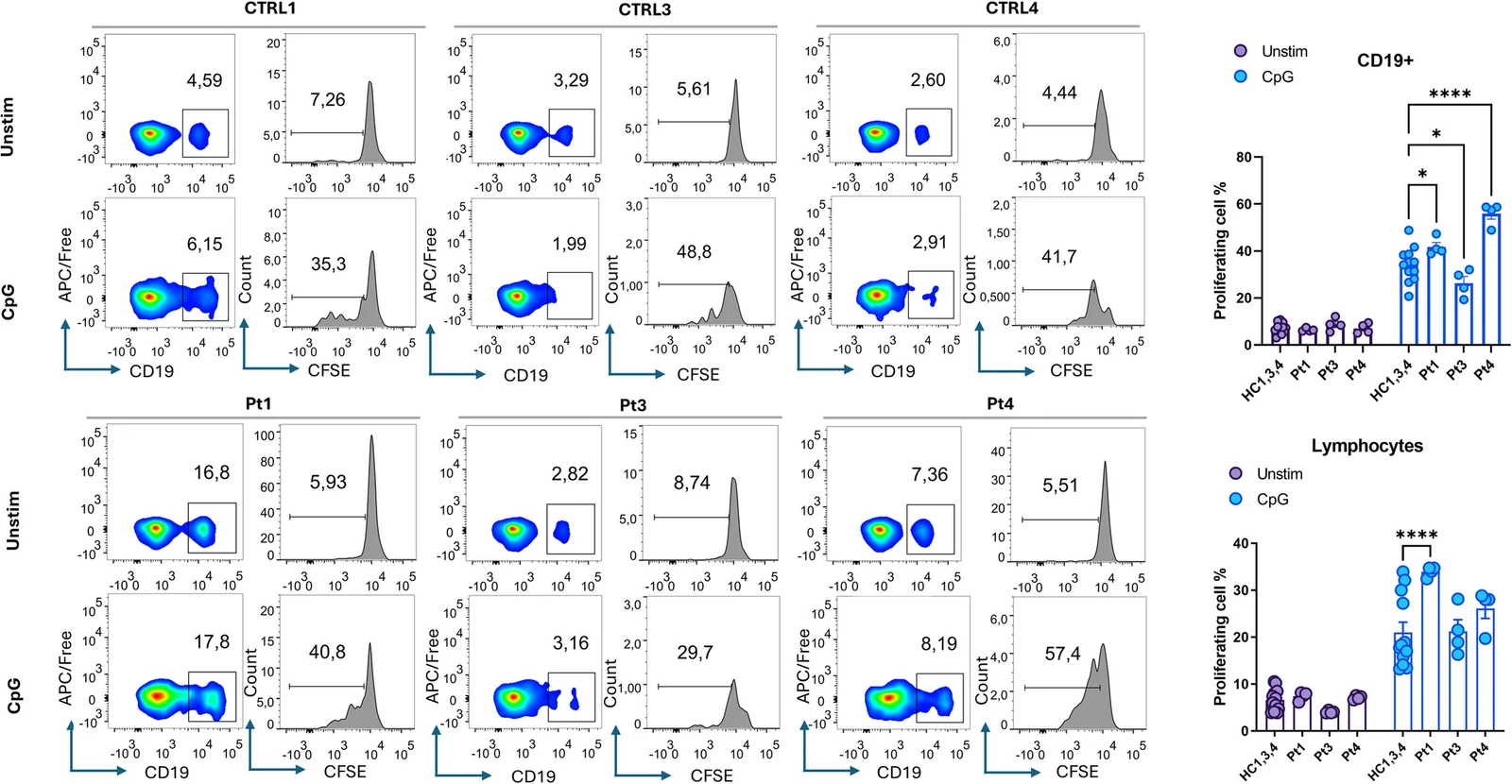
Proliferative capacity of lymphocytes and CD19⁺ B cells following CpG stimulation. Representative flow cytometry plots and cumulative data showing the proliferative response in healthy controls (CTRL1, 3, 4) and patients (Pt1, Pt3, Pt4). Representative pseudocolor plots and CFSE dilution histograms for unstimulated (Unstim.) and CpG-stimulated conditions are shown in left. The numbers indicate the percentage of gated cells. Bar graphs represent the percentage 1 of proliferating cells among total lymphocytes and the CD19⁺ B cell population. Statistical analysis was performed using ANOVA followed by Tukey’s multiple comparison test to compare stimulated patients against the stimulated healthy control (HC1,3,4) group. Significance is indicated as: *p < 0.05, ****p < 0.0001

Functional assays focused on B cell proliferation following CpG stimulation revealed that Pt1 and Pt4 had significantly higher proliferation rates compared to healthy controls (*p* < 0.05 and *p* < 0.0001, respectively), whereas Pt3 showed a significant reduction. Regarding general lymphocyte proliferation, Pt1 again showed a significant increase, while the responses in Pt3 and Pt4 remained stable. Overall lymphocyte and CD19⁺ B cell proliferation profiles are presented in Fig. 2.

## Discussion

B cell maturation involves dynamic surface marker changes, enabling the identification of distinct peripheral blood subpopulations via multicolor flow cytometry. Since the impaired formation of these subsets is a hallmark of various primary immunodeficiencies (PIDs), analyzing these stages provides critical insights into immune dysfunction. In this context, our findings are in total alignment with the established literature, demonstrating a uniform accumulation of B cells at the naive (CD27^−^ IgD^+^) stage and a profound loss of class-switched memory B cells (CD27^+^ IgD^−^) across all patients, regardless of the underlying genetic mutation (*CD40L* or *AICDA*) [12, 13]. However, a granular analysis of plasmablast and precursor distributions reveals critical differences in the biological mechanisms underlying these mutations, serving as a useful indicator for differential diagnosis.

In type 1 HIGM (CD40L deficiency), the lack of T-cell-dependent activation signaling prevents the initiation of the germinal center (GC) reaction [6]. In Pt 1, diagnosed with Type 1 HIGM (CD40L deficiency), the proportion of CD19^+^ B cells within total lymphocytes remains elevated, yet the qualitative integrity of these cells is severely compromised. This results in a maturation arrest at the naive stage, characterized by the total absence of switched memory B cells and plasmablast-like cells (CD20 − CD38++). Memory B cells are categorized into two distinct subsets based on the expression of CD27 and IgD. CD27⁺IgD⁺ memory B cells are thought to develop independently of the germinal center (GC) reaction and represent circulating marginal zone B cells that respond to T-independent antigens. In contrast, CD27⁺IgD⁻ (switched) memory B cells are products of the post-germinal center reaction. Consequently, a marked reduction or absence of these switched memory B cells is highly indicative of a disrupted GC reaction [14]. Due to the lack of CD40L-mediated T-cell help, these B cells are unable to initiate GC formation, which essentially halts antigen-specific memory development [15]. The absence of CD27^+^ and IgD^−^CD27^+^ (switched memory) cells points toward a maturation arrest at the naive stage [16]. Furthermore, the total lack of plasmablast-like cells (CD20^−^CD38^++^) suggests that terminal differentiation cannot proceed without functional CD40L signaling.

Conversely, in Type 2 HIGM (AID deficiency; Pt 2, 3, and 4), the CD40/CD40L interaction remains intact, allowing B cells to form GCs and express the CD27 surface marker. However, the absence of the AID enzyme renders nuclear CSR and somatic hypermutation biochemically impossible [8]. This leads to a “pseudo-maturation” state where B cells phenotypically appear mature by expressing CD27, yet remain functionally incapable of producing switched antibody isotypes. The landscape in Type 2 HIGM (*AID* deficiency), observed in Pt2, Pt3, and Pt4, is more complex. In these individuals, the CD40/CD40L interaction remains intact, enabling B cells to form GCs and acquire limited CD27 expression. While these cells may initiate GC reactions, the marked decrease of IgD^−^ CD27^+^ cells highlights their inability to successfully switch antibody classes [17].

When the double-negative (DN; CD27^−^IgD^−^) B cell subset was analyzed, a significant reduction was observed in Pt1 and Pt3, whereas this decrease did not reach statistical significance in Pt2 and Pt4. According to the literature, the DN1 subpopulation is considered a product of an aborted GC reaction and a precursor to switched memory (CD27^+^) B cells. In contrast, DN2 and DN3 cells are known to originate via GC-independent, extrafollicular pathways. The non-significant results in Pt2 and Pt4 suggest that the extrafollicular pathway might remain partially active or allow for a low level of DN cell production despite the underlying genetic defects. Furthermore, it is established that DN B cells tend to expand with aging and chronic infections [18]. Therefore, in Pt4—the oldest patient in our cohort—years of cumulative antigenic exposure may have driven the expansion of this subpopulation despite the genetic constraint, potentially through these alternative developmental routes.

The distinct precursor distributions further delineate the two HIGM types. In Pt1, the increase in CD38 + CD27− precursors alongside a plasmablast deficit reflects the absolute failure of GC formation due to CD40L deficiency. Conversely, the expansion of CD24^+^CD38^+^ populations in Pt2 suggests that AID-deficient B cells are driven toward maturation by antigenic stimulation but remain sequestered in a “pre-switched” or late-naive state. The increased plasmablast levels and high transitional cell (CD24^+^CD38^+^) proportions in Pt2 reflect a state where GCs can form but become hyperplastic. In alignment with our findings, a study in the literature involving 18 patients with Type 2 HIGM reported 13 lymphoid hyperplasia leading to tonsillectomy in 5 of these cases [19]. In a previous study, lymphoid hyperplasia was observed in 20 out of 29 patients prior to IVIG replacement therapy, with peripheral lymph nodes and tonsils being the most commonly affected sites. Similarly, Pt2 in our group presented with recurrent cervical lymphadenopathy and a history of adenotonsillectomy at age 3, suggesting an early-onset lymphoproliferative phenotype. While data in the literature often point to follicular hyperplasia with giant GCs, the clinical progression in our case—including follow-up by pediatric oncology—highlights the importance of monitoring these patients for lymphoproliferative complications [20]. This environment pushes B cells toward a plasmablast phenotype, yet they remain incapable of producing functional, switched antibody classes.

Peripheral B cell ontogeny progresses along a linear maturation spectrum defined by the dynamic shifts in CD38 and CD24 expression density. According to the model by Buffa et al., newly emigrated CD38^high^CD24^high^ (Transitional) cells gradually reduce these markers to become CD38^int^CD24^int^ (Mature Naive) and eventually CD38^−^ memory cells. The marked expansion of the CD24^+^CD38^+^population in Pt2 suggests a “maturational stasis“ [21]. Viewed through the functional compartmentalization theory of Sanz et al., this finding supports the notion that while these cells receive antigenic signals, genetic constraints (AID deficiency) prevent the completion of the GC reaction, causing them to accumulate in a “pre-memory” phase [22]. Patients with homozygous AICDA mutations (Pt2, Pt3, and Pt4) in our study exhibit a distinct biological pattern. In these cases, CD38⁺CD27⁻ and CD38⁺CD27⁺ populations remain comparable to healthy controls. This observation indicates that, unlike in CD40L deficiency (Pt1), the T-cell-dependent activation signaling is functionally preserved in AICDA-deficient patients.

Despite sharing the same homozygous AICDA mutation (c.70C>T, p.R24W), Pt2, Pt3, and Pt4 exhibit significant phenotypic inconsistencies. This clinical variation is most evident when comparing the two sisters (Pt2 and Pt3); while both share a history of recurrent respiratory tract infections (RTI), Pt2’s clinical course was marked by more severe lymphadenopathy and a history of adenotonsillectomy. These clinical disparities are further mirrored in the patients’ cellular profiles and proliferative capacities. Despite a significantly reduced CD19 + B cell frequency (Fig. 1), Pt4—the oldest in the cohort—exhibited the highest serum IgG levels. Furthermore, this patient showed a notably robust proliferative response to CpG stimulation (Fig. 2). This suggests that while the absolute B cell pool is contracted in Pt4, the remaining cells retain a high functional capacity for activation. In contrast, the cellular profiles of Pt2 and Pt3 show a different correlation. Pt3 exhibited both diminished peripheral B cell counts and a significantly lower proliferative response, indicating a more profound exhaustion or defect in her B cell compartment compared to Pt4. These variations highlight that peripheral B cell frequency alone does not always predict functional competency in Hyper-IgM patients, as compensatory proliferative mechanisms or disease chronicity may influence the immunophenotypic profile differently across individuals.

The proliferation data obtained in this study reveal the heterogeneous functional impact of different genetic mutations on B cell activation in HIGM. While CD40L deficiency is characterized by a lack of T-cell-dependent stimulation, it is well-established that these B cells can remain responsive to T-cell-independent signals, such as the TLR9 ligand CpG. The increased proliferation observed in Pt1 suggests that the TLR9 activation pathway is not only preserved but may also exhibit a compensatory hyper-responsiveness in the absence of CD40L-mediated signaling. Imai et al. demonstrated that B cells from both HIGM2 and HIGM4 patients are capable of achieving normal proliferation when stimulated in vitro with sCD40L and IL-4 [23]. Our results using CpG stimulation are consistent with this observation, as Pt1 (CD40L deficiency) and Pt4 (AICDA mutation) exhibited significantly increased proliferation rates compared to healthy controls (*p* < 0.05 and *p* < 0.0001, respectively). This is consistent with the hypothesis that HIGM B cells can remain highly responsive to activation stimuli, even when the downstream terminal differentiation and class-switching machinery is defective. Interestingly, a notable divergence was observed between Pt3 and Pt4 despite both sharing a homozygous *AICDA* mutation. While Pt4 showed robust proliferation, Pt3 exhibited significant suppression. This suggests that AID deficiency may involve broader signaling or survival defects that vary among individuals, extending beyond simple class switching. These findings suggesting that compensatory immune mechanisms or environmental exposures may partially mitigate the AICDA defect over time.

The clinical presentation of our study group is highly consistent with the established literature on primary B cell immunodeficiencies. As highlighted in the previous study, recurrent RTIs represent the most frequent clinical manifestation in these patients [20, 24]. In total alignment with these reports, recurrent upper RTIs were the primary clinical finding across our entire patient group [20]. De la Morena et al. suggest that even stable Type 1 HIGM patients remain at risk for life-threatening complications. Therefore, stem cell transplantation should be considered the definitive treatment [25]. For Type 2 HIGM patients, the indications for stem cell transplantation are less standardized compared to Type 1. Specifically, our first patient (*CD40L* deficiency), who is continuing IVIG therapy and antimicrobial prophylaxis, is stable with the current treatment; not performing a transplant in this patient is consistent with a more conservative management approach.

In conclusion, detailed analysis of B cell subsets elucidates the fundamental biological disparities between HIGM subtypes. The significant loss of CD27^+^IgD^−^ cells and the mutation-specific shifts in maturation stages highlight the critical importance of flow cytometry in the diagnosis and clinical monitoring of HIGM.

## Limitations

Several limitations of this study should be acknowledged. First, the sample size is relatively small, which is a common challenge in research involving rare primary immunodeficiencies. While this limited cohort size may constrain the generalizability of our findings to all HIGM subtypes, it reflects the clinical reality of managing such rare conditions. Second, our immunophenotypic analysis was restricted to peripheral blood mononuclear cells (PBMCs), which may not fully represent the complex B-cell dynamics and germinal center reactions occurring within secondary lymphoid organs. Third, we observed significant inter-individual phenotypic and functional variability, particularly among patients with the same homozygous *AICDA* mutation. A key limitation is the lack of additional parameters, such as detailed environmental exposure history or epigenetic profiling, which might have better elucidated the underlying causes of this clinical and proliferative heterogeneity. Despite these limitations, this study has yielded significant findings in the field of B-cell immunopathology. Specifically, through high-resolution, multi-parameter flow cytometric analysis, we compared X-linked CD40L deficiency with autosomal recessive homozygous AICDA mutations (c.70C>T; p.R24W). We extended our characterization beyond basic subsets to include detailed differentiation stages, such as transitional cells and plasmablasts. This approach allowed for the identification of mutation-specific ‘pseudo-maturation’ states and maturation arrest points, which may serve as critical indicators for differential diagnosis. Furthermore, the inclusion of functional B cell proliferation assays highlights the heterogeneous effect of different genetic defects on B cell activation, offering valuable insights into disease mechanisms and potential diagnostic strategies for HIGM patients. However, further studies with larger sample sizes are recommended to confirm these results.

## Data Availability

No datasets were generated or analysed during the current study.
